# On-Treatment NLR Dynamics During CDK4/6 Inhibition Are Associated with Overall Survival in Metastatic Breast Cancer

**DOI:** 10.3390/cancers18172894

**Published:** 2026-09-07

**Authors:** Baha Sharaf, Zaid Omari, Qasem Alzoubi, Anas Zayed, Faris Tamimi, Ahmad Khater, Maen Hamad, Sharif Jehad, Nader Obeidat

**Affiliations:** 1 King Hussein Cancer Center, Amman 11941, Jordan; zo.11516@khcc.jo (Z.O.); qa.13630@khcc.jo (Q.A.); az.12421@khcc.jo (A.Z.); ft.10481@khcc.jo (F.T.); ahmadkhatter46@gmail.com (A.K.); mh.17723@khcc.jo (M.H.); naderobeidat001@gmail.com (N.O.); 2 Faculty of Medicine, Yarmouk University, Irbid 21163, Jordan; shareefsharaf456@gmail.com

**Keywords:** neutrophil-to-lymphocyte ratio, CDK4/6 inhibitor, ribociclib, metastatic breast cancer, landmark analysis, tumor microenvironment, biomarker

## Abstract

Background/Objectives: In metastatic breast cancer (MBC) treated with CDK4/6 inhibitors, baseline neutrophil-to-lymphocyte ratio (NLR) is an established prognostic marker. On-treatment NLR dynamics have shown inconsistent associations with survival, and no prior study has applied a dominant-driver decomposition to classify patients by whether NLR change is neutrophil- or lymphocyte-driven. Methods: We retrospectively analyzed 352 patients with HR+/HER2− MBC treated with ribociclib. Using paired neutrophil and lymphocyte counts at baseline and at approximately 12 weeks (before cycle 4), a log-linear decomposition classified patients into four NLR-trajectory phenotypes by the dominant driver of change. Associations with progression-free survival (PFS) and overall survival (OS) were assessed using a 4-month landmark approach with multivariable Cox models, with consistency evaluated across four thresholds, tertiles, and a continuous model. Secondarily, NLR change was compared across five response-trajectory groups. Results: NLR trajectory was not associated with PFS in any specification (multivariable hazard ratio [HR] 1.15 per standard deviation [SD], 95% CI 0.97–1.37, *p* = 0.12) but was independently associated with OS (HR 1.40 per SD, 95% CI 1.18–1.67, *p* < 0.001), confirmed on bootstrap resampling. Adding NLR trajectory improved discrimination (C-index +0.04) and fit (likelihood-ratio *p* < 0.001). The OS effect was time-varying, attenuating beyond 24 months. NLR trajectory was unrelated to dose-limiting neutropenia (*p* = 0.58) or dose reduction (*p* = 0.69). Primary refractory patients showed blunted NLR decline versus responding or stable patients (*p* = 0.005), independent of baseline NLR. Conclusions: On-treatment NLR trajectory is a correlate of OS, independent of PFS, dose-limiting neutropenia, and dose reduction, in ribociclib-treated MBC, distinct from direct tumor control. Prospective validation is warranted.

## Simple Summary

A simple blood test measuring the neutrophil-to-lymphocyte ratio (NLR) reflects the balance between pro-inflammatory and immune-protective cells. Although baseline NLR predicts survival in hormone-driven metastatic breast cancer treated with ribociclib, it is unknown whether NLR changes during treatment carry prognostic information. We studied 352 patients and measured NLR at baseline and after approximately 12 weeks of treatment (before cycle 4). How NLR changes during treatment predicted overall survival but not cancer progression, and it was unrelated to drug side effects. Patients with primary refractory disease showed almost no NLR decline. These results suggest NLR dynamics reflect the patient’s overall immune fitness rather than a direct antitumor effect of the drug. These preliminary findings require confirmation in larger prospective studies.

## 1. Introduction

Breast cancer is the most frequently diagnosed cancer and a leading cause of cancer death among women worldwide [1]. Cyclin-dependent kinase 4/6 inhibitors (CDK4/6i) combined with endocrine therapy are standard treatment for hormone receptor-positive (HR+), HER2-negative metastatic breast cancer (MBC) [2,3]. These agents act by blocking phosphorylation of the retinoblastoma (Rb) protein, arresting tumor cells in the G1 phase of the cell cycle [4], though acquired resistance to this mechanism remains common and clinically important [5]. Randomized trials of ribociclib, the CDK4/6 inhibitor used exclusively in this cohort [6,7], have demonstrated a significant overall survival benefit when added to endocrine therapy in both the first-line (MONALEESA-2) [2] and later-line (MONALEESA-3) [3] settings.

NLR serves as a low-cost, peripheral readout of the delicate, opposing forces in the cancer-immunity dynamic. Neutrophilia is largely driven by the tumor itself. By secreting cytokines like G-CSF, GM-CSF, IL-6, and IL-8/CXCL8, tumors actively expand and recruit neutrophils. These cells infiltrate the tissue as tumor-associated neutrophils (TANs) and, once inside the established breast tumor microenvironment (TME), predominantly polarize toward a pro-tumor phenotype. Rather than fighting the cancer, these TANs promote angiogenesis, suppress cytotoxic CD8+ T-lymphocyte function, and recruit myeloid-derived suppressor cells (MDSCs) to help the tumor evade the immune system. Lymphopenia represents the loss of the body’s active immune defense against the tumor. When combined with the systemic stress caused by the cancer, this drop in lymphocytes explains why a high NLR is not entirely tumor-specific; it also reflects physical stress.

Ultimately, a high NLR in the blood mirrors a suppressed immune environment inside the tumor itself. Across different cancers, a higher NLR directly correlates with a weakened immune state, associating with fewer cancer-fighting CD8+ T-cells and more immunosuppressive cells in pancreatic cancer [8], as well as a depleted profile of tumor-infiltrating lymphocytes (TILs) in lung cancer [9].

These TILs are incredibly critical. In breast cancer, their presence is a strong, independent predictor of better survival, a relationship clearly proven even in aggressive triple-negative disease [10].

Because of these dynamics, baseline NLR has been studied more extensively in breast cancer than in almost any other solid tumor.

Early data quickly established its clinical value. An initial meta-analysis of 12 studies linked a high baseline NLR to significantly shorter disease-free survival (DFS; HR 1.46, 95% CI 1.12–1.90) and overall survival (OS; HR 2.03, 95% CI 1.41–2.93) [11]. A subsequent meta-analysis of 15 studies involving 8,563 patients confirmed these findings, showing comparable pooled risks (OS HR 2.56, 95% CI 1.96–3.35; DFS HR 1.74, 95% CI 1.47–2.07). However, this larger analysis also highlighted a key challenge: substantial variation in NLR cutoff values (ranging from 1.9 to 5.0), driven partly by differences in tumor stage, receptor status, and grade [12]. Since then, even larger meta-analyses have expanded on these findings, collectively evaluating more than 17,000 patients [13,14].

We are also beginning to see how molecular subtypes influence this relationship. While one analysis identified NLR as an independent adverse marker, specifically in luminal A disease [15], these subtype-specific effects are not yet consistently reproduced across different patient cohorts and cutoffs. Meanwhile, the clinical utility of NLR continues to expand into targeted therapies. Baseline NLR is now recognized as an independent prognostic marker for progression-free survival (PFS) and OS across multiple CDK4/6 inhibitor cohorts [16,17], and a large real-world series recently linked it directly to a patient’s risk of developing severe neutropenia [16].

While baseline measurements are well established, far fewer studies have explored how NLR changes during treatment, and the existing data point in conflicting directions. In patients treated with CDK4/6 inhibitors for metastatic breast cancer, one cohort showed a highly unexpected result where a decrease in related inflammatory ratios during the first cycle of therapy was paradoxically linked to shorter survival [18]. This finding directly challenges the straightforward clinical assumption that greater drug-induced myelosuppression represents higher drug exposure and ultimately better tumor control. Conversely, in the distinct clinical setting of triple-negative breast cancer where CDK4/6 inhibitors are not used, dynamic NLR shifts behave in a much more predictable manner. An initial cohort of 329 patients demonstrated that changes in NLR during therapy are strongly and independently prognostic of outcomes [19]. Furthermore, a larger analysis tracking 600 triple-negative breast cancer patients across four time points found that when NLR rose rather than fell, the risk of recurrence or death climbed significantly, showing an increased risk of 12% to 20% per assessment interval [20]. Mirroring this trend, patients with lower pretreatment NLRs also achieved significantly higher pathologic complete response rates following neoadjuvant chemotherapy [21]. In MBC specifically, evidence is more conflicting still: a 2024 meta-analysis of 29 NLR studies and 12 absolute-lymphocyte-count (ALC) studies found greater heterogeneity than in early-stage cohorts [22], and a single-center Spanish cohort of 263 MBC patients found NLR prognostic on univariate analysis but not independently significant after multivariable adjustment [23]. This directional and contextual inconsistency across studies has not been resolved, no study has separated the neutrophil (numerator) from the lymphocyte (denominator) contribution to NLR change to test whether doing so clarifies the picture, and no study has related on-treatment NLR change to two independent time-points of response assessment within the same cohort.

We addressed these gaps in a real-world MBC cohort with paired baseline/4-month blood counts, dose-limiting-neutropenia and dose-reduction flags, and 3-month plus best/last response data, using landmark methodology and pre-specified sensitivity analyses to test the stability of any signal identified.

## 2. Materials and Methods

### 2.1. Study Population

This retrospective, single-center cohort comprised 352 consecutive patients with HR+/HER2− MBC who received ribociclib in combination with endocrine therapy (aromatase inhibitor, 89.8%; fulvestrant, 10.2%). All patients were female. This registry has previously been reported in studies examining treatment outcomes [6] and the prognostic impact of HER2-low expression [7] in this patient population. Absolute neutrophil and lymphocyte counts were extracted at baseline and at approximately 12 weeks (before cycle 4 of ribociclib; the pre-cycle 4 routine assessment was the scheduled time-point for NLR measurement in our protocol) on treatment. No neutrophil or lymphocyte count of zero was encountered, so the log-linear decomposition was defined for all patients with available paired counts. Dose reduction was recorded as a binary indicator. OS event was defined as death during follow-up; PFS event was defined as the earlier of documented disease progression (best/last response coded ‘DP’) or death.

### 2.2. NLR Trajectory Phenotype Derivation

Among the 352 patients, 283 (80.4%) had paired absolute neutrophil (N) and lymphocyte (L) counts available at baseline and at the on-treatment assessment. The on-treatment sample was the scheduled pre-cycle 4 blood count, obtained approximately 12 weeks after initiation. Because every on-treatment count was therefore drawn before the 4-month landmark, the exposure was fully ascertained at the time origin of the survival analyses and no measurement contributed information from after the landmark. For clarity, “on-treatment NLR” refers throughout to this approximately 12-week measurement, and “4-month landmark” refers to the time origin for survival analysis. Of these 283 patients, three died between the on-treatment blood draw and the 4-month landmark and were therefore not eligible for the overall survival landmark cohort (n = 280); a further 16 had documented progression before the landmark and were additionally ineligible for the progression-free survival landmark cohort (n = 264). For these patients, proportional changes in neutrophil and lymphocyte counts were calculated using natural-log transformations. The corresponding change in the neutrophil-to-lymphocyte ratio (NLR) was derived as the difference between the log changes in neutrophils and lymphocytes (Δlog[NLR] = Δlog[N] − Δlog[L]), allowing changes in the NLR to be attributed to the relative contributions of each cell type.

Patients were classified into four mutually exclusive trajectory phenotypes: NLR increase (≥10% increase from baseline), stable NLR (<10% increase or decrease), neutrophil-driven decrease (≥10% decrease in which |Δlog[N]| > |Δlog[L]|, i.e., the change in neutrophil count contributed more to the ratio movement), or lymphocyte-driven decrease (≥10% decrease in which |Δlog[L]| > |Δlog[N]|, i.e., the change in lymphocyte count contributed more to the ratio movement than the change in neutrophil count). All other ≥10% decreases, in which |Δlog[N]| ≥ |Δlog[L]|, were classified as neutrophil-driven.

The ±10% threshold for defining NLR stability was specified a priori to reduce misclassification caused by normal analytical and biological variability around zero change. This magnitude approximates the reported day-to-day within-subject coefficient of variation of automated differential leukocyte counts (on the order of 8–10%), so that changes smaller than the threshold are unlikely to represent true on-treatment shifts [19,20]. Because no consensus cutoff for dynamic NLR exists in the CDK4/6-inhibitor setting, we did not anchor the threshold to any single prior study; instead its influence on classification was examined across ±5%, ±15%, and ±20% reclassifications and a threshold-free tertile analysis (Results).

### 2.3. Landmark Analysis

Because NLR trajectory was defined using on-treatment blood counts, including it as a baseline covariate in a conventional Cox model would introduce immortal time bias, as patients had to survive on treatment for their trajectory phenotype to be determined [24,25]. To address this, we performed a 4-month landmark analysis as described by Anderson et al. [26], with survival time redefined from the landmark date. Patients were required to be alive and progression-free at 4 months for the progression-free survival (PFS) analysis, whereas only survival to 4 months was required for the overall survival (OS) analysis. Separate eligibility criteria were used because patients who progressed before 4 months remained eligible for the OS analysis if they were still alive. This resulted in a PFS landmark cohort of 264 patients (146 subsequent progression events) and an OS landmark cohort of 280 patients (122 subsequent deaths).

### 2.4. Statistical Analysis

Continuous variables are presented as median (interquartile range [IQR]) and categorical variables as number (percentage). Progression-free survival (PFS) and overall survival (OS), measured from the respective 4-month landmark, were estimated using the Kaplan–Meier method and compared across NLR trajectory phenotypes using the log-rank test.

Separate multivariable Cox proportional hazards models were fitted for the PFS and OS landmark cohorts. The primary exposure was NLR trajectory phenotype (reference: stable NLR), adjusted for baseline NLR (≥2.5 vs. <2.5), ECOG performance status (0–1 vs. ≥2), visceral disease, line of CDK4/6 inhibitor therapy (first, second, or later), and menopausal status. Complete-case analysis was used for missing covariates.

Because the stable-NLR group was small (n = 16–20), two pre-specified sensitivity analyses were performed by changing the reference category to the NLR-increase group and, separately, to a combined stable/increase group. To evaluate the robustness of the predefined ±10% stability threshold, trajectory phenotypes were reclassified using ±5%, ±15%, and ±20% thresholds and, independently, by tertiles of the continuous Δlog[NLR] distribution. As an additional sensitivity analysis, Δlog[NLR] was modelled as a standardized continuous variable rather than as a categorical phenotype.

The proportional hazards assumption was assessed using Schoenfeld residuals. When violations were identified, models were re-fitted separately during the early and late follow-up periods, divided at the cohort median. Internal validation of the continuous Δlog[NLR] OS model was performed using 500 bootstrap resamples, reporting the bootstrap mean, median, 95% percentile interval of the hazard ratio, and the proportion of resamples with an effect estimate in the same direction as the original model.

Incremental prognostic value was assessed by changes in Harrell’s concordance index (C-index), Akaike information criterion (AIC), and the likelihood ratio test.

Associations between NLR trajectory phenotype and clinically documented dose-limiting neutropenia or treatment dose reduction were evaluated using the χ^2^ test or Fisher’s exact test, as appropriate. In secondary analyses, patients were assigned to five response-trajectory groups by combining the 3-month response assessment with best overall response (RECIST 1.1): primary refractory (progression at 3 months); stable followed by progression (stable disease at 3 months, progression as best response); early response followed by late progression (partial or complete response at 3 months, progression as best response); sustained responder (partial or complete response at 3 months maintained as best response); and stable-sustained (stable disease at 3 months maintained as best response). Δlog[NLR] was compared across these five groups using the Kruskal–Wallis test, followed by Bonferroni-adjusted Mann–Whitney U tests for pairwise comparisons. Effect modification of baseline NLR by visceral disease was evaluated by including a multiplicative interaction term in a Cox model fitted to the full non-landmarked cohort. No adjustment was made for multiplicity across the threshold, tertile, subgroup, and response-trajectory analyses; apart from the primary landmark models, all analyses are exploratory and the associated *p* values are descriptive.

All statistical tests were two-sided, with *p* < 0.05 considered statistically significant for the primary analyses. Analyses were performed using Python 3.12 (pandas, lifelines, and scipy).

## 3. Results

### 3.1. Cohort

Median age was 52.1 years (IQR 44.0–61.9); 182/351 (51.9%) had visceral disease (one patient had missing visceral status); 223 (63.4%) received ribociclib as first-line therapy (Table 1). Median baseline NLR was 2.15 (IQR 1.57–3.19). Median follow-up was 41 months (IQR 27–62); 211 PFS events and 160 deaths occurred; median PFS was 26 months and median OS 40 months.

### 3.2. NLR Trajectory and PFS

Among the 283 evaluable patients, 202 (71.4%) had a neutrophil-driven decrease in NLR, 43 (15.2%) an NLR increase, 19 (6.7%) stable NLR, and 19 (6.7%) a lymphocyte-driven decrease. In the PFS landmark cohort (n = 264; 146 events), NLR trajectory phenotype was not associated with progression-free survival in either unadjusted (log-rank *p* = 0.36) or adjusted analyses (continuous Δlog[NLR]: HR per SD 1.15, 95% CI 0.97–1.37; *p* = 0.12; Table 2). Visceral disease (HR 1.72–1.78; *p* = 0.002) and receipt of CDK4/6 inhibitor therapy beyond the second line (HR approximately 3.6; *p* < 0.001) were the only independent predictors of shorter PFS.

### 3.3. NLR Trajectory and OS

**Figure 1:**
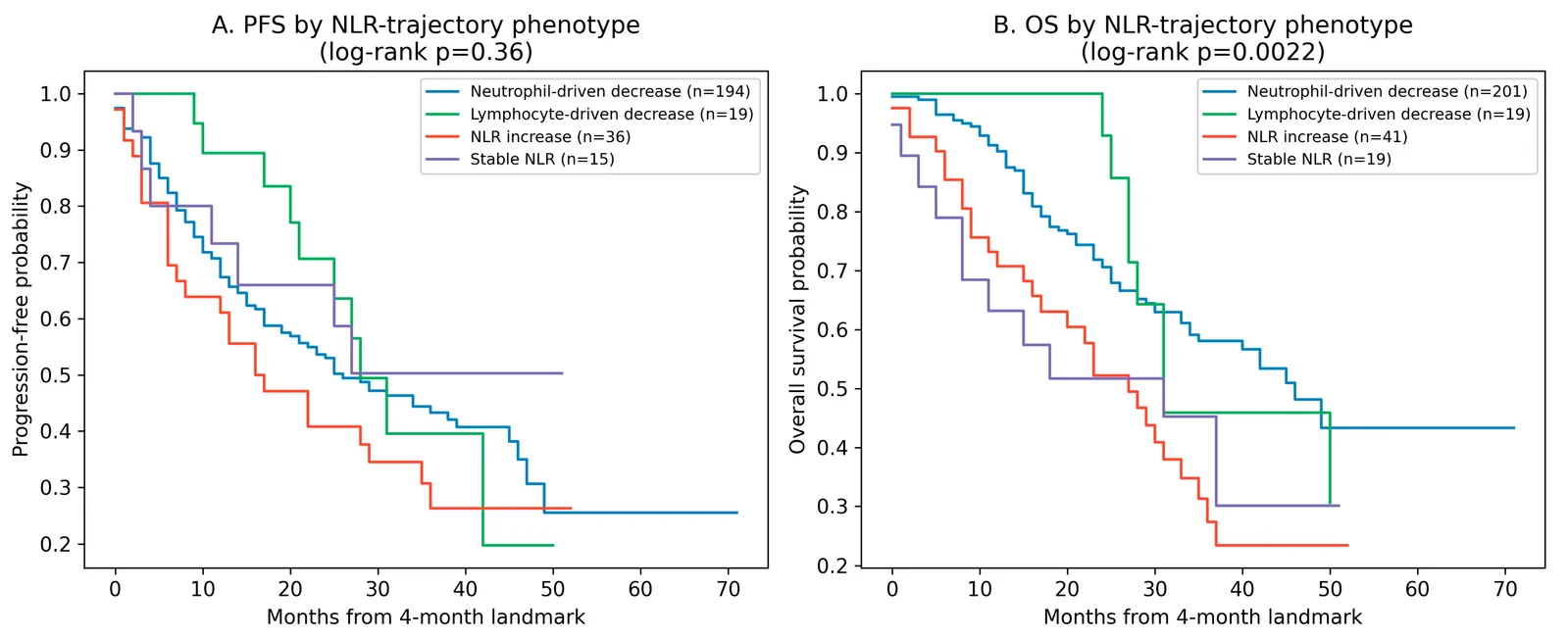
Kaplan-Meier survival curves from the 4-month landmark by NLR-trajectory phenotype. (**A**) Progression-free survival (PFS; n = 264, 146 events). (**B**) Overall survival (OS; n = 280, 122 deaths). NLR, neutrophil-to-lymphocyte ratio.

In the OS landmark cohort (n = 280; 122 deaths), NLR trajectory phenotype was significantly associated with overall survival (log-rank *p* = 0.0022). In the adjusted Cox model (n = 275; 120 deaths), both a neutrophil-driven decrease (HR 0.44, 95% CI 0.23–0.84; *p* = 0.01) and a lymphocyte-driven decrease (HR 0.35, 95% CI 0.14–0.88; *p* = 0.03) were associated with a lower hazard of death than stable NLR, whereas the NLR-increase phenotype was not (HR 0.88, 95% CI 0.43–1.80; *p* = 0.72) (Figure 1; Table 2).

**Figure 2:**
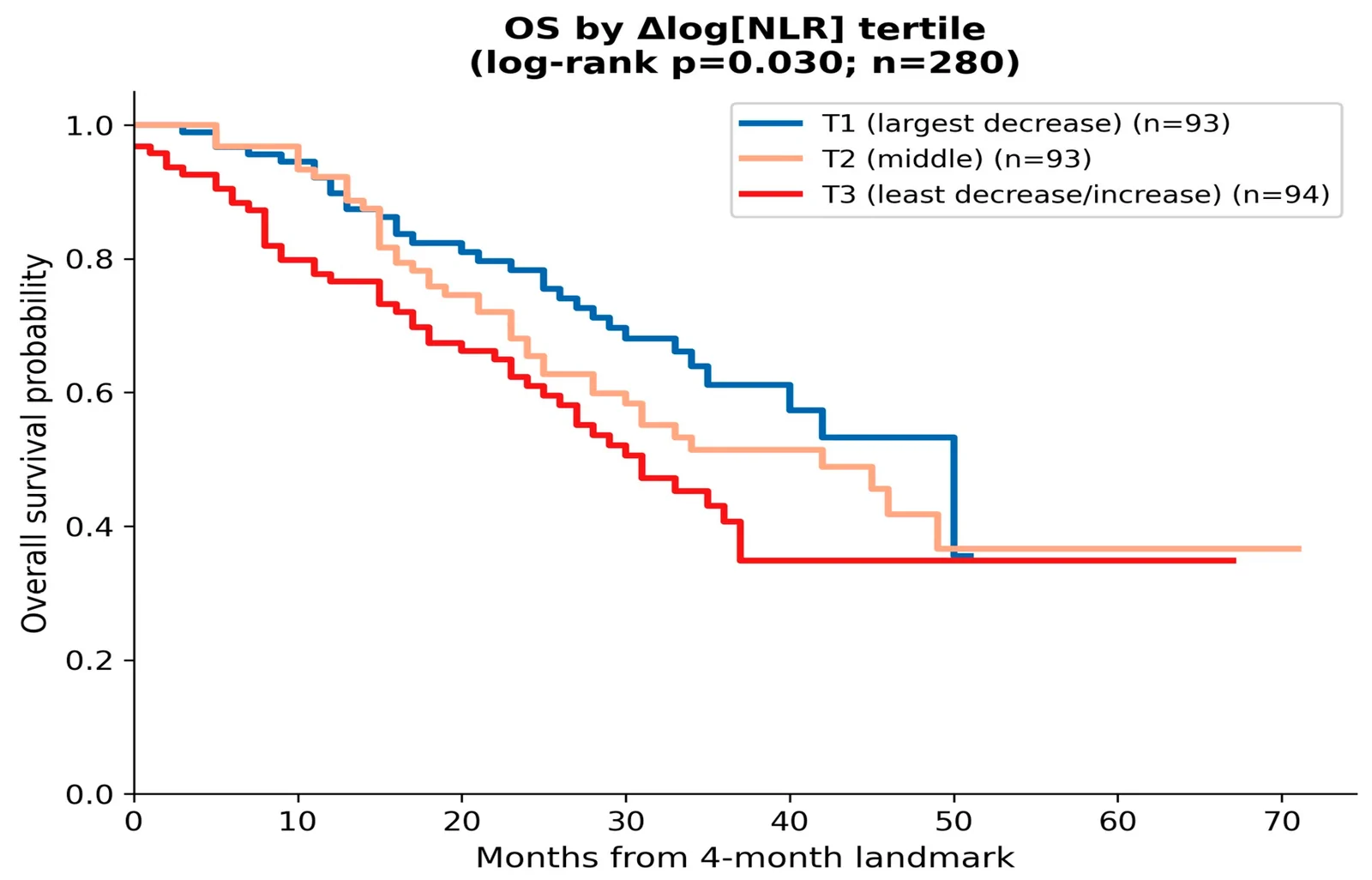
OS from the 4-month landmark by data-driven Δlog[NLR] tertile (n = 280; log-rank *p* = 0.030). T1 = largest decrease, T2 = middle, T3 = increase or smallest decrease. Δlog[NLR], log-linear change in the neutrophil-to-lymphocyte ratio.

These findings were supported by the continuous Δlog[NLR] analysis (HR per SD 1.40, 95% CI 1.18–1.67; *p* < 0.001). The association with OS remained significant across all predefined stability thresholds (±5%, ±10%, ±15%, and ±20%; log-rank *p* = 0.002–0.014) and in a threshold-free tertile analysis, which demonstrated a monotonic gradient in median OS (50, 42, and 31 months; log-rank *p* = 0.030) (Figure 2). In contrast, no consistent association was observed with PFS, with significance reached only at the widest stability threshold (±20%; *p* = 0.041). NLR trajectory phenotype was not associated with dose-limiting neutropenia (*p* = 0.58) or treatment dose reduction (*p* = 0.69). The distribution of the four phenotypes shifted predictably as the stability window widened, with the stable-NLR group enlarging at the expense of the two decrease phenotypes: at ±5% the phenotypes comprised neutrophil-driven 205 (72%), lymphocyte-driven 19 (7%), increase 46 (16%), and stable 13 (5%); at ±10% (primary definition) they comprised 202 (71%), 19 (7%), 43 (15%), and 19 (7%); at ±15% they comprised 195 (69%), 18 (6%), 40 (14%), and 30 (11%); and at ±20% they comprised 188 (66%), 18 (6%), 34 (12%), and 43 (15%) (all n = 283 evaluable). The neutrophil-driven decrease remained the dominant phenotype at every threshold, and the OS association persisted throughout, indicating the result is not an artefact of the specific ±10% cutoff.

### 3.4. Sensitivity Analysis Restricted to Progression-Free Patients

To exclude the possibility that the overall survival association was driven by the 16 patients who had already progressed by the four-month landmark, the continuous OS model was repeated in the 264 patients who were alive and progression-free at the landmark (identical to the PFS cohort). The association was confirmed: HR 1.28 per standard deviation of Δlog[NLR] (95% CI 1.05–1.56, *p* = 0.014; n = 258 complete-case, 105 deaths).

### 3.5. Reference-Group Sensitivity, Model Diagnostics, and Validation

Because the stable-NLR reference group was small (n = 16–20), we performed pre-specified sensitivity analyses using alternative reference categories. Re-fitting the OS model with the NLR-increase group as the reference, and subsequently with a combined stable/increase group, produced nearly identical hazard ratios and statistical significance, confirming that the association between NLR trajectory phenotype and OS was robust to reference-group selection (Table 3).

The proportional hazards assumption was satisfied for all covariates in the PFS model but was violated for NLR trajectory phenotype in the OS model. Exploratory time-stratified analyses showed that the survival benefit associated with neutrophil- and lymphocyte-driven decreases was greatest during the first 24 months after the landmark and attenuated thereafter, indicating that the overall hazard ratios represent time-averaged effects.

Internal validation using 500 bootstrap resamples yielded results consistent with the primary model, with a mean hazard ratio of 1.44 (median 1.42; bootstrap 95% percentile interval 1.21–1.73), closely matching the original estimate (HR 1.40, 95% CI 1.18–1.67). In all bootstrap samples, the hazard ratio remained greater than 1. Adding continuous Δlog[NLR] to the baseline clinical model improved both discrimination (Harrell’s C-index, 0.626 to 0.665) and model fit (AIC, 1186.3 to 1175.0; likelihood ratio χ^2^ = 13.3, 1 degree of freedom, *p* < 0.001) (Table 3).

**Table 2:** Full multivariable Cox models, 4-month landmark, complete-case (PFS n = 259/142 events, concordance 0.62; OS n = 275/120 events, concordance 0.67). Complete-case reduction from the respective landmark cohorts (n = 264 PFS, n = 280 OS) is due to missing ECOG performance status (n = 5 in each); all other covariates were complete. For the OS model, the trajectory-phenotype hazard ratios are time-averaged: the proportional-hazards assumption was violated for this variable (Schoenfeld *p* = 0.02 for neutrophil-driven and *p* = 0.001 for lymphocyte-driven decrease), and the effect attenuates beyond 24 months from the landmark; see time-split analysis in Table 3.

Covariate	PFS HR (95% CI), *p*	OS HR (95% CI), *p*
Baseline NLR ≥ 2.5	1.16 (0.81–1.65), 0.42	1.40 (0.96–2.06), 0.08
Neutrophil-driven decrease (vs. stable)	1.19 (0.54–2.61), 0.66	0.44 (0.23–0.84), 0.01
Lymphocyte-driven decrease (vs. stable)	0.80 (0.29–2.20), 0.67	0.35 (0.14–0.88), 0.03
NLR increase (vs. stable)	1.58 (0.67–3.70), 0.29	0.88 (0.43–1.80), 0.72
ECOG ≥ 2 (vs. 0–1)	1.35 (0.62–2.98), 0.45	1.93 (0.92–4.05), 0.08
Visceral disease	1.72 (1.22–2.41), 0.002	1.68 (1.15–2.45), 0.007
Line beyond 2nd (vs. 1st)	3.59 (2.23–5.81), <0.001	3.70 (2.23–6.14), <0.001
Line 2nd (vs. 1st)	1.23 (0.82–1.85), 0.31	1.27 (0.81–1.98), 0.30
Premenopausal (vs. postmenopausal)	0.83 (0.59–1.18), 0.30	0.84 (0.58–1.22), 0.36

**Table 3:** Reference-group sensitivity, proportional hazards diagnostics, and internal validation for the OS model.

Analysis	Result
Reference = increase phenotype (n = 41)	Neutrophil-driven decrease HR 0.50 (0.31–0.79), *p* = 0.003; lymphocyte-driven decrease HR 0.40 (0.18–0.89), *p* = 0.03
Reference = stable + increase combined (n = 60)	Neutrophil-driven decrease HR 0.48 (0.32–0.73), *p* < 0.001; lymphocyte-driven decrease HR 0.38 (0.17–0.84), *p* = 0.02
Schoenfeld residuals, OS model	Violated for phenotype (neutrophil-driven *p* = 0.02; lymphocyte-driven *p* = 0.001); held for all other covariates
Early vs. late follow-up split (median 24 mo)	0–24 months: HR 1.42 (95% CI 1.16–1.75, *p* = 0.001); >24 months: HR 1.24 (95% CI 0.87–1.79, *p* = 0.236)
500-sample bootstrap, continuous ΔNLR OS model	Mean HR 1.44, 95% percentile CI 1.21–1.73; all bootstrap resamples HR > 1
Discrimination/fit, adding ΔNLR to baseline model	C-index 0.626→0.665; AIC 1186.3→1175.0; LR *p* < 0.001

### 3.6. Visceral Disease as Effect Modifier

In the full cohort (n = 317), the interaction between baseline NLR and visceral disease was not significant (HR 1.07, 95% CI 0.80–1.42, *p* = 0.66). Exploratory subgroup analyses of continuous Δlog[NLR] (hazard ratio per SD, OS) showed consistent direction across visceral disease status (visceral: HR 1.26, 95% CI 1.01–1.57, *p* = 0.041; non-visceral: HR 1.84, 95% CI 1.35–2.52, *p* < 0.001) and treatment line (first-line: HR 1.33, 95% CI 1.00–1.77, *p* = 0.048; later-line: HR 1.38, 95% CI 1.12–1.70, *p* = 0.002).

### 3.7. Response-Trajectory Discordance

**Figure 3:**
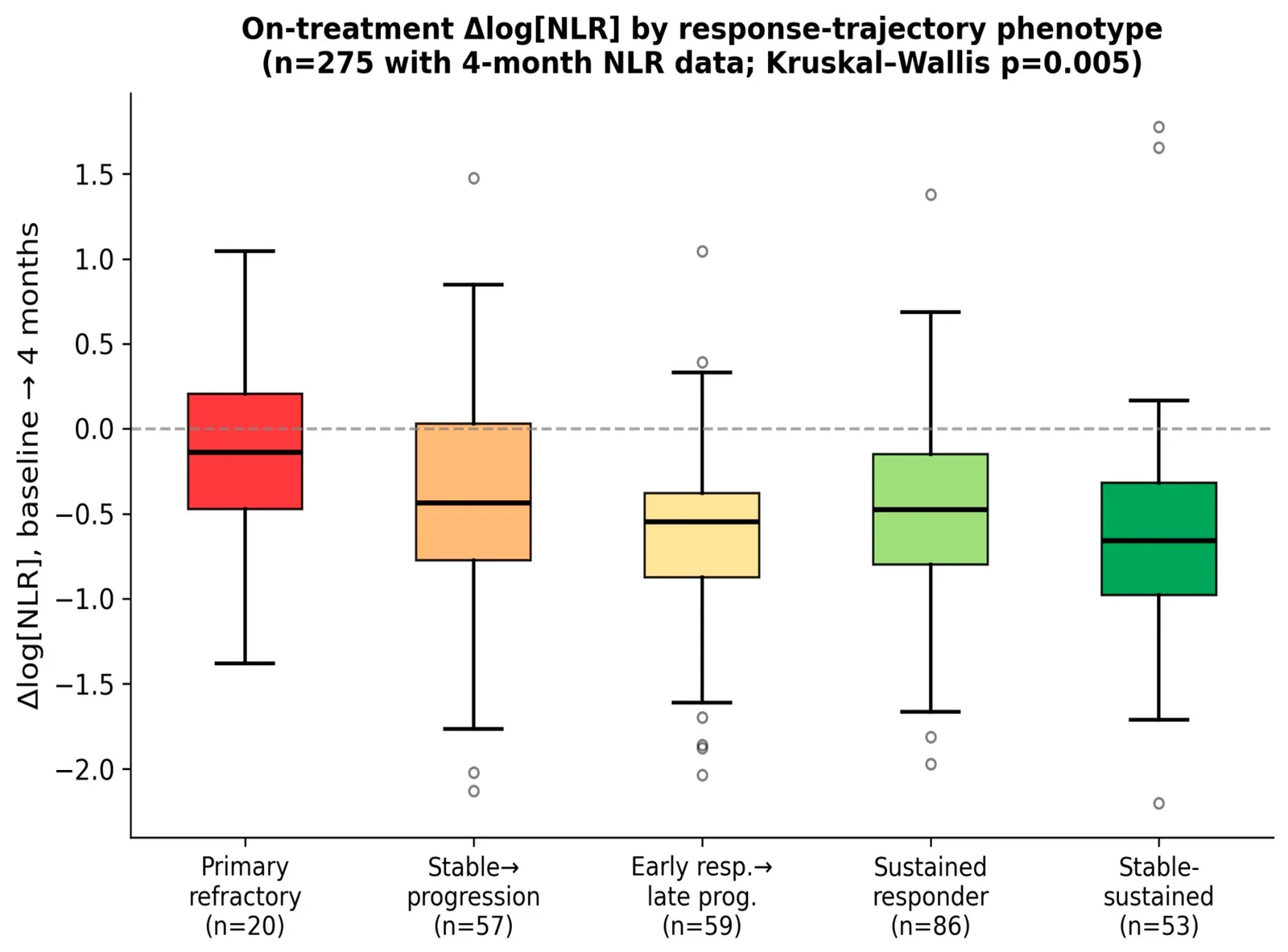
On-treatment Δlog[NLR] by response-trajectory discordance phenotype (n = 275 with 4-month NLR data; Kruskal–Wallis *p* = 0.005). Δlog[NLR] = log-linear change in NLR; dashed line indicates Δlog[NLR] = 0.

Response trajectories were classifiable in 275 patients with available 4-month NLR data: primary refractory (n = 20), stable followed by progression (n = 57), early response followed by late progression (n = 59), sustained responder (n = 86), and stable-sustained (n = 53). On-treatment Δlog[NLR] differed significantly across these groups (Kruskal–Wallis *p* = 0.005; Figure 3), with the smallest decline observed in primary refractory disease and larger, broadly similar declines in the four other groups. Baseline NLR did not differ across the same groups (Kruskal–Wallis *p* = 0.529), indicating that the signal reflects on-treatment change rather than pretreatment inflammatory burden. As Δlog[NLR] and the three-month response assessment were obtained at the same clinical visit (before cycle 4, approximately 12 weeks after initiation), this analysis cannot establish the temporal relationship between NLR dynamics and early resistance, and the finding should be considered as hypothesis-generating.

## 4. Discussion

This analysis set out to resolve a directional inconsistency in the dynamic-NLR literature by log-linearly decomposing NLR change into neutrophil- and lymphocyte-driven components in a CDK4/6i-treated MBC cohort, and to test the consistency of any signal identified across multiple analytic specifications rather than a single arbitrary cutoff.

### 4.1. The Primary Finding: An OS-Specific, PFS-Independent Signal

The decomposed categorical NLR-trajectory phenotype did not predict PFS at any specification tested but was independently and dose-dependently associated with OS across six operationalizations (four thresholds, tertiles, and a continuous exposure). Bootstrap validation and improved discrimination and fit on adding Δlog[NLR] to the baseline clinical model (C-index +0.04; AIC −11; likelihood ratio *p* < 0.001; Table 3) indicate this is not an artifact of an arbitrarily chosen cutoff or reference category. Proportional-hazards testing showed the effect is concentrated in the first two years from the landmark and attenuates thereafter, so the reported hazard ratio should be read as a time-averaged effect rather than a constant one.

This OS-specific dissociation, which was not explained by dose-limiting neutropenia or dose reduction, is mechanistically informative: if NLR decline reflected primarily on-target ribociclib-induced marrow suppression, we would expect the signal in PFS and a correlation with dose-limiting neutropenia; neither occurred. Consistent with this, the association with OS was not explained by dose-limiting neutropenia or dose reduction (dose-limiting neutropenia *p* = 0.58; dose reduction *p* = 0.69), indicating that the NLR-trajectory signal is not simply a surrogate of myelosuppressive drug exposure. This pattern instead parallels the phase 3 EMBRACE trial of eribulin (a microtubule inhibitor, not a CDK4/6i), in which NLR behaved as a general prognostic marker of host status across treatment arms rather than a drug-specific predictive biomarker [27]. These findings suggest the host-status interpretation is not confined to any one drug class, consistent with the CDK4/6i-specific glucose-to-lymphocyte ratio findings of Yilmaz and colleagues [17]. The underlying biological mechanism remains uncertain and cannot be resolved from these data. A direct comparison of the categorical neutrophil-driven and lymphocyte-driven decrease groups in the OS model (HR 0.44 vs. HR 0.35, both vs. stable NLR) showed overlapping confidence intervals (*p* for between-group comparison = 0.73), indicating that the two subgroups cannot be statistically distinguished from each other with the current sample sizes. The value of the decomposition is therefore primarily methodological: it confirms the OS signal is driven by decline in either cellular component and is not an artefact of lymphocyte count alone. The PD-1/PD-L1 axis is a key regulator of lymphocyte activity captured in the NLR denominator; checkpoint-driven lymphocyte exhaustion reduces circulating lymphocyte counts and elevates NLR independently of neutrophil dynamics [28]. When CDK4/6 inhibitors reduce tumor proliferation, reduced tumor-derived immunosuppressive signaling may partially restore lymphocyte counts, contributing to NLR decline [28]. Additionally, mast cell-derived mediators including IL-8 and TNF-α promote neutrophil recruitment to the tumor microenvironment and suppress effector lymphocyte activity, directly influencing both components of NLR [29]. As CDK4/6 inhibitors alter the inflammatory cytokine milieu, mast cell activity may be one intermediary through which systemic NLR dynamics are modulated.

Our qualitative direction, in which a falling NLR was favorable and a rising NLR unfavorable, is directionally concordant with the TNBC dynamic-NLR literature and general breast-cancer meta-analyses discussed above [11,12,13,19,20,21] but contrasts with the one existing CDK4/6i-specific dynamic-ratio study, which found a paradoxical, opposite-direction association over a shorter (4-week), differently composed (platelet/monocyte-based) window [18]. The opposing direction of that finding is best understood through a systematic comparison of the two cohorts rather than the biomarker window alone: Moukas et al. sampled at a single 4-week timepoint using platelet- and monocyte-based ratios in a cohort not restricted by landmark survival, whereas our analysis used a four-month landmark, a neutrophil/lymphocyte decomposition, and overall survival as the primary endpoint, with substantially longer follow-up. Differences in cohort stage composition, distribution of treatment lines, endpoint definition, and follow-up length are therefore all plausible contributors to the divergent conclusions, and no single factor can be isolated as the sole explanation from the available data. These cross-subtype comparisons also warrant caution because the tumor immune microenvironments of HR+/HER2− and triple-negative breast cancer differ materially: triple-negative tumors are generally more immunogenic, with higher tumor-infiltrating lymphocyte density and greater PD-L1 expression, whereas HR+/HER2− tumors are typically immunologically colder. A peripheral ratio such as NLR may therefore reflect different aspects of host–tumor immune interaction in the two subtypes, and prognostic patterns observed in triple-negative cohorts should not be assumed to transfer directly to the HR+/HER2− setting studied here. Kim CG and colleagues, studying CDK4/6 inhibitor-treated hormone-receptor-positive MBC, reported that a derived NLR measured during therapy was associated with progression-free survival [30]; this contrasts with our null PFS finding. The divergence is most plausibly explained by differences in design: Kim et al. analyzed a derived NLR at a single early on-treatment timepoint without a landmark, whereas we applied a four-month landmark and a dominant-driver decomposition that classifies each patient by whether the NLR change was neutrophil- or lymphocyte-driven. These distinct analytical targets, an early absolute value versus a decomposed trajectory conditioned on surviving to the landmark, can yield different endpoint associations, and this constitutes a specific and testable target for external validation. Baseline NLR itself was not independently significant in either fully adjusted model (Table 2), consistent with a single-center Spanish MBC cohort that similarly found NLR’s prognostic value substantially confounded by other factors [23], though this diverges from larger, mostly early-stage meta-analyses [11,12,13].

### 4.2. The Response-Trajectory Finding

The response-trajectory analysis provides a complementary, more PFS-proximal signal: primary refractory patients (progression by 3 months) showed significantly blunted NLR decline independent of baseline NLR. Because both were assessed at the same clinical visit, this remains hypothesis-generating. This is consistent with persistence of an immunosuppressive, myeloid-dominant TME despite CDK4/6 inhibition [8] and extends the baseline-NLR-as-resistance-marker literature [19,20,23] by suggesting the early trajectory, not just the starting value, may better separate primary resistance from response. To our knowledge this has not previously been reported in CDK4/6i-treated MBC.

### 4.3. Strengths and Limitations

Strengths: This is, to our knowledge, the first study to log-linearly decompose on-treatment NLR change into neutrophil- and lymphocyte-driven components in CDK4/6i-treated MBC and the first to relate on-treatment NLR trajectory to an independent, two-timepoint response-discordance phenotype in this treatment setting. The finding was stress-tested across six alternative operationalizations of the same Δlog[NLR] exposure (which are correlated rather than independent), two alternative reference-group choices, 500-sample bootstrap resampling, and incremental discrimination/fit metrics rather than relying on a single model specification. Event numbers were adequate for the multivariable models presented (142 PFS and 120 OS events in the respective landmark cohorts).

Limitations: This study is retrospective and single-center, with unblinded, non-centrally reviewed response assessments. Because all patients received ribociclib, the findings may not be generalizable to other CDK4/6 inhibitors, such as palbociclib or abemaciclib, which have different hematologic toxicity profiles. The attribution of NLR changes to neutrophil or lymphocyte dynamics was based on paired measurements at baseline and 4 months rather than serial assessments, limiting evaluation of temporal changes. Although the 4-month landmark approach was necessary to avoid immortal time bias, it excluded patients who progressed or died before the landmark and therefore conditioned all survival analyses on surviving to that time point. Two trajectory groups remained relatively small, reducing the precision of subgroup-specific estimates despite consistent findings in sensitivity analyses. The proportional hazards assumption was not met for the trajectory phenotype in the OS model, indicating that its prognostic effect varied over time and that the reported hazard ratios represent average effects across follow-up. Missing covariate data were handled using complete-case analysis, and potential confounders affecting circulating neutrophil and lymphocyte counts, including infection, corticosteroid use, and granulocyte colony-stimulating factor administration, were not available. Additionally, residual confounding from unmeasured clinical or biological factors cannot be excluded. The Ki-67 proliferation index was not available for all patients; data availability was systematically related to diagnosis era rather than random missingness (routine Ki-67 testing was discontinued during the study period), precluding a reliably adjusted Ki-67 sensitivity analysis. In addition, because NLR incorporates the neutrophil count, we cannot fully exclude that subclinical, non-dose-limiting treatment-related changes in neutrophils contributed to the observed NLR dynamics; however, the absence of any association with dose-limiting neutropenia or dose reduction argues against myelosuppression being the principal driver. Finally, although the association between NLR trajectory and OS remained consistent across multiple prespecified sensitivity analyses, these findings do not establish causality and require external validation before clinical application.

## 5. Conclusions

On-treatment NLR trajectory was consistently associated with overall survival, but not progression-free survival, in patients with ribociclib-treated metastatic breast cancer across multiple prespecified analytic approaches. In addition, the failure of NLR to decline early during treatment was associated with primary refractory disease. These findings are consistent with previous studies of dynamic NLR in breast cancer and with evidence that peripheral NLR reflects the tumor immune environment. However, they are based on a single retrospective cohort and should be considered hypothesis-generating. Prospective external validation, ideally incorporating serial blood sampling, is needed before these observations can be translated into clinical practice. Accordingly, no treatment recommendations can be made on the basis of the present findings.

## Figures and Tables

**Table 1:** Cohort characteristics (n = 352 unless otherwise noted). Denominators below 352 reflect missing baseline data: visceral status (n = 1 missing) and ECOG performance status (n = 8 missing).

Characteristic	Value
De novo metastatic disease	75 (21.3%)
Sex, female	352 (100%)
Age, years, median (IQR)	52.1 (44.0–61.9)
Postmenopausal	188 (53.4%)
ECOG ≥ 2	15/344 (4.4%)
Visceral disease	182/351 (51.9%)
First-line Ribociclib	223 (63.4%)
Baseline NLR, median (IQR)	2.15 (1.57–3.19)
Dose-limiting neutropenia	75 (21.3%)
Dose reduction	100 (28.4%)
Median follow-up, months (IQR)	41 (27–62)
PFS events/median PFS	211 (59.9%)/26 mo
Deaths/median OS	160 (45.5%)/40 mo

## Data Availability

The datasets generated during and/or analyzed during the current study are available from the corresponding author on reasonable request.
